# Molar incisor hypomineralization: proportion and severity in primary public school children in Graz, Austria

**DOI:** 10.1007/s00784-017-2150-y

**Published:** 2017-06-19

**Authors:** Barbara Buchgraber, Lumnije Kqiku, Kurt A. Ebeleseder

**Affiliations:** University Clinic of Dental Medicine and Oral Health, Division of Prosthodontics, Restorative Dentistry, Periodontology and Implantology, Medical University Graz Austria, Billrothgasse 4, 8010 Graz, Austria

**Keywords:** MIH, Proportion, Enamel hypomineralization, Developmental defects

## Abstract

**Objective:**

The aim of this study was to determine the proportion and severity of molar incisor hypomineralization (MIH) in primary school children in Graz (southeast of Austria).

**Materials and methods:**

In 1111 children aged 6 to 12 years (mean age 9.0 ± 1.2), a wet examination of all teeth was performed by three trained examiners using a dental chair, optimal illumination, a dental mirror, and a dental explorer. All teeth with MIH lesions were registered so that different definitions of MIH were applicable. According to the European Academy of Pediatric Dentistry criteria that were considered valid at the time of the investigation, MIH was diagnosed when at least one first primary molar (FPM) was affected.

**Results:**

MIH was present in 78 children (7.0%). In 64 children (5.8%), at least one molar *and* one incisor were affected (so-called M + IH). Additionally, in 9 children, only incisors were affected. In 7 affected children, teeth other than FPMs and incisors had MIH lesions. Almost an equal number of males (38) and females (40) were affected. The upper and lower molars were equally affected. The upper incisors were more frequently affected than the lower ones. Demarcated enamel opacities were the predominant types of defects.

**Conclusion:**

The proportion of MIH was 7.0% in Graz, which is similar to other comparable trials.

**Clinical relevance:**

This study has proven that MIH is an existing dental problem in Graz.

## Introduction

During the last 15 years, molar incisor hypomineralization (MIH) has been gaining attention in pediatric dentistry. MIH clinically presents in incisors and the first permanent molars (FPMs) as demarcated enamel opacities of a different color. Its severity differs not only between patients but also within the mouths of the patients, and its appearance can be asymmetrical. Due to soft and porous enamel, more severely affected teeth can undergo post-eruptive breakdown of hard tissue. This can lead to crown destruction or atypical restorations frequently ending in tooth loss [1]. Increased sensitivity of the affected teeth can make children afraid of brushing their teeth and of dental treatment. Therefore, European dentists consider MIH to be a clinical problem [1].

According to the policy document from the European Academy of Pediatric Dentistry (EAPD), MIH is diagnosed if at least one first permanent molar is affected by demarcated opacity, enamel breakdown, or atypical restoration on the occlusal and buccal surfaces [2, 3]. There is a wide variation in the proportion of MIH throughout the world, ranging from a low proportion in China at 2.5% [4] to a high proportion in Brazil at 40.2% [5]. In Austria’s neighboring countries, the prevalence varies from 14.4% in Slovenia [6], 13.7% in Italy [7], 6.4% in Switzerland [8], and 5.8% in Germany [9]. In Western Austria, one study [10] reported an MIH prevalence of 13.5% in the county of Salzburg and of 8.0% in the county of Tyrol (overall prevalence of 10.9% in a sample of 1283 children from second- and third-grade classes) [10]. In this study, a dry inspection was performed using a pocket lamp. Males had a higher proportion of MIH than females (55.7:44.3), and in 48.6% of all MIH-positive cases, only one FPM was affected. The buccal aspects of the FPMs (67.9%) were more frequently affected than the occlusal (62.1%), lingual (46.3%), and approximal (16.7%) ones.

MIH studies from the eastern part of Austria are still lacking. The aim of this study was to determine the proportion and severity of MIH in primary school children in Graz (southeast of Austria).

## Materials and methods

Approval for this investigation was obtained from the Ethics Committee of the Medical University of Graz, and consent was obtained from the children that were selected for the study and their parents (Ethics Protocol No: 21-134 ex 09/10).

Graz is located in Styria, a county in the southeast of Austria. Its 262,566 inhabitants make it Austria’s second largest city. Its drinking water contains a low fluoride level (<0.1 mg/l). A dental health program (DHP) has been installed by the City Office of Education in public primary schools. Among others, the main goals of the DHP are to conduct a yearly oral inspection of every child to visually detect caries and orthodontic treatment needs and to provide a report of these results to the parents including recommendations for treatment. MIH is not included in the DHP. To carry out the DHP, the City Office of Education employs three dentists on a full-time basis, each of them in a separate school dental clinic. These three dentists (A, B, and C) were asked to include the diagnosis of MIH in the DHP. They agreed to do so, but the City Office of Education did not give us permission to use data other than those relating to MIH.

Primary school education in Austria comprises 4 years (grades) and starts when children are 6 years old. It is organized into classes with 15–35 children per class.

At the time of the investigation, there were 50 primary schools in Graz, including 38 public schools comprising 6938 pupils and 12 private schools comprising 1828 pupils. The latter were not available for this study.

A sample size of 1000 children was estimated to be statistically sufficient, which translated to 250 children from each grade and 334 children per investigator.

Classes were selected according to the investigation schedule of the DHP. Further randomization was not possible in the daily working schedule of the DHP.

Three to four classes per investigator and grade were needed to reach the desired number of children. Overall, 15 schools were assigned to the three school dental clinics (sdc-A, sdc-B, sdc-C) and were then invited to take part in the study in collaboration with the DHP. None of the schools refused. One thousand two hundred two first- to fourth-grade primary school children were asked to participate. None of the pupils refused to participate, as the examination was part of the DHP. The only reasons for children to be excluded from participation were if they did not fulfill the inclusion criteria or if they matched the exclusion criteria.

Inclusion criteria were as follows:

- Between ages 6 and 12 (born in 1998–2003)
- Presence of sufficiently erupted molars (at least one molar erupted more than half of the crown)

Exclusion criteria were as follows:

- Consent form could not be obtained from the parents
- Child’s absence on the day of the examination

Ninety-one children were not included; thus, in total, 1111 (547 males:564 females) first- to fourth-grade primary school children were examined. The distribution of the investigated children according to classes, grades, and examiners is documented in Table 1. The mean age of the children at the time of the examination was 9.0 ± 1.2 years.

**Table 1:** Age, gender, and distribution of the investigated children. Class grades and examiners

Examiner	A	B	C	Total
1st-grade children/classes	107/5	80/4	84/5	271/14
2nd-grade children/classes	113/5	77/4	94/5	284/14
3rd-grade children/classes	78/4	80/4	103/5	261/13
4th-grade children/classes	92/4	109/5	94/5	295/14
Total	390/18	346/17	375/20	1111/55

All clinical examinations were performed at the three school dental clinics. Before the examination, all participating children received a single-use toothbrush with fluoridated toothpaste to use to clean their teeth under the supervision of the examiner. Next, the children were placed in a dental chair. Using a halogen light, dental mirrors, and dental probes, the three previously trained dentists (see below for training details) performed a full-mouth inspection of the wet teeth. Thus, all teeth were investigated for the presence of MIH lesions.

So as not to disrupt the schedule of the DHP, the three investigators made a simple yes/no decision regarding the presence of MIH in a particular tooth. To educate parents, each affected child was given information on MIH and was invited for a second examination (performed by the author BB at the Department of Conservative Dentistry) in which the severity grade according to the criteria of the EAPD was evaluated for all affected teeth.

In the DHP, caries lesions were scored and documented on a separate data sheet, but as mentioned previously, these records were not available for this study due to lack of permission.

According to the EAPD guidelines [2], children were to be diagnosed as affected by MIH if at least one FPM was affected by hypomineralization, enamel breakdown, or atypical restoration [3]. MIH opacities in teeth other than molars and incisors were noted. Isolated lesions in incisors without involvement of FPMs were also noted but were not classified as MIH cases.

Furthermore, the following findings were not diagnosed as MIH: demarcated opacities of less than 1 mm in diameter, amelogenesis imperfecta, tetracycline staining, erosion, white spot caries lesions, and fluorosis. Prior to the study, the examiners had received training in the diagnosis of MIH, carried out by the author BB in the following format: a 1-h PowerPoint presentation was given on MIH (according to the EAPD criteria: at least one FPM affected) and confusable enamel defects. Colored handouts were given for personal study use. One week later, knowledge was verified through brief oral communication.

Calibration was then achieved through the use of 29 clinical photographs (18 cases of MIH and 11 of non-MIH), which came from the author BB. A kappa statistic was used to measure the concordance between the 3 examiners and the diagnoses of MIH and produced kappa values of 0.78, 0.85, and 0.93.

Statistical analysis was carried out in cooperation with the Institute of Medical Informatics, Statistics and Documentation of the Medical University of Graz. The collected data were analyzed using SPSS package version 19.0 (SPSS Inc., Chicago, IL, USA). A descriptive analysis of the proportion and distribution of MIH was performed. A correlation of the affected teeth with the jaws was analyzed using a Pearson chi-square test. Fisher’s exact test was performed to analyze the severity. A level of *p* < 0.05 was considered statistically significant.

## Results

According to the EAPD criteria, MIH was present in 78 (7.0%; 95% CI 5.5–8.6%) of the 1111 children, including 38 males and 40 females (48.7% male; 51.3% female). The breakdown of MIH diagnosis by group is as follows: 33 children in sdc-A (42.3%), 16 children in sdc-B (20.5%), and 29 children in sdc-C (37.2%). Fourteen children (17.9%) presented with hypomineralized FPMs only, whereas 64 (82.1%) children presented with at least one hypomineralized molar and at least one hypomineralized incisor (so-called M + IH [3] = 5.76%) Additionally, nine subjects with only MIH lesions in their permanent incisors were found but were not diagnosed as having MIH. Seventy-four (94.9%) affected children had all of their FPMs erupted. Sixty-one (78.2%) affected children had all of their incisors erupted. Among the 74 children with all four of their FPMs erupted, 28 (35.7%) had all of their FPMs affected, 18 (23.1%) had 3 FPMs affected, 12 (16.7%) had 2 FPMs affected, and 16 (24.4%) had only a single one affected. The distribution of all of the affected FPMs in all 78 children by school grade is documented in Table 2.

**Table 2:** Number of affected FPMs distributed by grade

	1 affected molar	2 affected molars	3 affected molars	4 affected molars	Total	Affected FPMs per affected child
1st-grade children	3 (3.8%)	3 (3.8%)	4 (5.1%)	11 (14.1%)	21 (26.9%)	3.10 ± 1.14
2nd-grade children	4 (5.1%)	2 (2.6%)	6 (7.7%)	6 (7.7%)	18 (23.1%)	2.18 ± 1.22
3rd-grade children	10 (12.8%)	2 (2.6%)	6 (7.7%)	4 (5.1%)	22 (28.2%)	2.24 ± 1.27
4th-grade children	2 (2.6%)	6 (7.7%)	2 (2.6%)	7 (9.0%)	17 (21.8%)	2.82 ± 1.13
Total	19 (24.4%)	13 (16.7%)	18 (23.1%)	28 (35.7%)	78 (100.0%)	2.55 ± 1.24

**Fig. 1:**
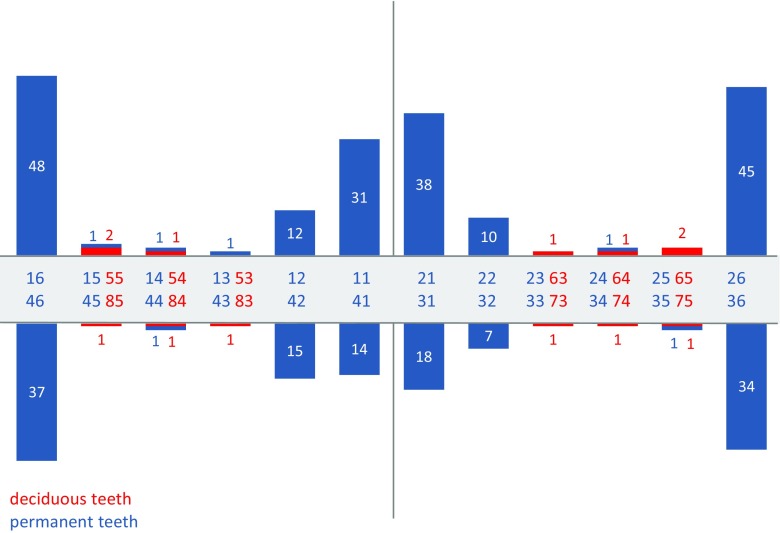
Distribution of MIH-affected teeth

The distribution pattern of all of the teeth with MIH lesions can be found in Fig. 1. Maxillary FPMs were more frequently affected than mandibular FPMs (53 vs. 44 children), but this difference was not statistically significant (Fisher’s exact test *p* = 0.081). Maxillary incisors were more frequently affected than mandibular incisors (43 vs. 29 children), with a statistically significant difference (Fisher’s exact test *p* = 0.018).

Severity grade in FPMs: From the total of 307 FPMs present in 78 affected children, 211 FPMs were affected with MIH, according to the criteria of the EAPD, and 164 were able to be reexamined to determine the severity grade. One hundred seventeen FPMs (71%) had demarcated opacities, 29 FPMs (18%) presented atypical restorations, and 18 FPMs (11%) were found to have post-eruptive enamel breakdown (Table 3). In this sample, three children had one or two FPMs extracted for unknown reasons. These missing teeth could not be identified as having been extracted due to MIH.

**Table 3:** MIH-affected PFMs and incisors according to the criteria of EAPD

	Demarcated opacity	Post-eruptive enamel breakdown	Atypical restoration	Total
All molars	117 (71.3%)	18 (11%)	29 (17.7%)	164 (100.0%)
All incisors	144 (99.3%)	0 (0.0%)	1 (0.7%)	145 (100.0%)

Severity grade in incisors: From the total of 572 incisors present in 78 affected children, 180 were affected, and 145 were able to be re-examined to determine the severity grade. Only one presented an atypical restoration, and all others were classified as demarcated opacities. Thus, incisors were generally less severely affected than molars. With more FPMs affected, the percentage of having at least one affected incisor was higher, but there was no statistical association (Fisher’s exact test *p* = 0.777). There was an average of 2.71 affected FPMs (211/78) and 2.31 affected incisors (180/78) per affected child.

MIH lesions in other teeth: In 7 children (9% of all affected children), there were teeth other than FPMs and incisors that were affected with MIH defects (*n* = 19), including primary canines (*n* = 3), primary molars (*n* = 10), permanent canines (*n* = 1), and premolars (*n* = 5).

## Discussion

This is the first internationally published study investigating the proportion of MIH in eastern Austria. With a sample size of 1111 children, the examined population may be large enough to compare with that of other studies [6–10]. The present study derived its sample from public primary schools only; therefore, it may not be considered to be socioeconomically representative. Based on this, and on shortcomings involved with randomization, our results are to be considered only as proportions and not as general prevalence.

Compared with other studies from Central Europe, the proportion of 7.0% was low. Prevalence data from other European countries seem to show a north-south inclination, with some Nordic countries showing a higher prevalence than those in Central Europe [11, 12]. The data from the current study and a second study from western Austria [10] fit loosely into this pattern. The proportion of MIH in Graz (7%) is close to the percentage reported in Switzerland (6.4% among 7–8-year-olds) [8]; however, these proportions are lower than the proportions in Italy (13.7%/7.3–8.3 years) [7]; Slovenia (14%/12–18 years) [6]; Munich, Germany (14.7%/10.2 years on average) [3]; Düsseldorf, Germany (14.6%/7.3–8.9 years) [13]; Northern England (15.9%/12 years on average) [14]; and Barcelona, Spain (17.85%/6–14 years) [15], but are somewhat higher than the proportion in Giessen, Germany (5.9%/6–12 years) [13].

In the present study, no significant difference was found between the number of affected maxillary and mandibular FPMs, which is in agreement with other studies [7, 16, 17]. Maxillary incisors were more frequently affected than mandibular ones, which has also been found in other studies [4, 9, 18]. Almost an equal number of males and females were affected, which is also in agreement with other studies [7, 9, 11, 12].

Severe MIH defects (atypical restorations or enamel disintegration) were more present in FPMs than in incisors. Several cross-sectional studies have shown that in general, the defects in incisors are mild [25, 26]. The involvement of incisors appears to increase with an increasing number of affected FPMs, which is in agreement with other studies [9, 11, 18, 19].

Similar to other studies [18, 20–22], most lesions in our investigation were mild demarcated opacities in both molars and incisors. Although the color of the enamel defects was not recorded in the present study, the overall impression was that most defects were creamy white as previously described [23, 24]. In the present study, 9% of MIH-affected children had teeth other than FPMs and incisors that were affected with MIH lesions. To understand the nature of MIH, it is of great importance to know the extent to which teeth other than FPMs and incisors are affected [25].

Children who had one to three unerupted FPMs were included. Although it was not specifically mentioned in the original criteria, we also included partially erupted teeth (more than half of the crown erupted) as suggested by Calderara et al. [7]. The diagnosis of MIH was based on the presence of at least one afflicted FPM, and only demarcated opacities of at least 1 mm in diameter were included [26]. These three issues helped to avoid over-registration but do not help to explain the low proportion of 7.0%.

Although it was not shown to be as prevalent as in other Central European countries, this study has proven that MIH is an existing dental problem in Graz. However, the present study does not represent the Austrian community as a whole. As demonstrated by Petrou et al. in Germany [13], the proportion of MIH may show wide regional variations in Austria also.

For this reason, additional regions of Austria should be investigated.

## Conclusion

The findings of this study indicate that MIH has a moderate proportion of 7% in the eastern part of Austria. Most of the affected children were mildly affected.
